# Sites responsible for infectivity and antigenicity on nervous necrosis virus (NNV) appear to be distinct

**DOI:** 10.1038/s41598-021-83078-3

**Published:** 2021-02-11

**Authors:** Hyun Jung Gye, Toyohiko Nishizawa

**Affiliations:** Department of Aqualife Medicine, Chonnam National University, Yeosu, 59626 Republic of Korea

**Keywords:** Viral proteins, Viral infection, Virus structures

## Abstract

Nervous necrosis virus (NNV) is a pathogenic fish-virus belonging to the genus *Betanodavirus* (*Nodaviridae*). Surface protrusions on NNV particles play a crucial role in both antigenicity and infectivity. We exposed purified NNV particles to different physicochemical conditions to investigate the effects on antigenicity and infectivity, in order to reveal information regarding the conformational stability and spatial relationships of NNV neutralizing-antibody binding sites and cell receptor binding sites. Treatment with PBS at 37 °C, drastically reduced NNV antigenicity by 66–79% on day one, whereas its infectivity declined gradually from 10^7.6^ to 10^5.8^ TCID_50_/ml over 10 days. When NNV was treated with carbonate/bicarbonate buffers at different pHs, both antigenicity and infectivity of NNV declined due to higher pH. However, the rate of decline with respect to antigenicity was more moderate than for infectivity. NNV antigenicity declined 75–84% after treatment with 2.0 M urea, however, there was no reduction observed in infectivity. The antibodies used in antigenicity experiments have high NNV-neutralizing titers and recognize conformational epitopes on surface protrusions. The maintenance of NNV infectivity means that receptor binding sites are functionally preserved. Therefore, it seems highly likely that NNV neutralizing-antibody binding sites and receptor binding sites are independently located on surface protrusions.

Nervous necrosis virus (NNV) is known to infect more than 120 fish species and causes high mortality in aquaculture facilities worldwide^1–3^. NNV, a member of the genus *Betanodavirus* in *Nodaviridae*, has a non-enveloped spherical shape with a diameter of 25–30 nm. It consists of a single coat protein (CP, *M*_r_ 42,000) and two molecules of positive-sense single-stranded RNA^4^. On the NNV particle surface, there are 60 protrusions formed by trimeric protrusion-domains (P-domain) of CP that play crucial roles in NNV antigenicity and infectivity^5–7^. Surface protrusions and other genomic aspects are involved in the virulence and host-specificity of NNV^8,9^. NNV neutralizing-antibodies are directed against conformational epitopes on surface protrusions^7,10^. At least three different serotypes of NNV have been reported based on neutralization tests^11,12^, although NNV are classified into four genotypes based on nucleotide sequences of the viral CP gene^13,14^. Surface protrusions are relatively unstable against moderate-low temperatures and/or carbonate/bicarbonate buffer^7,15^. Interestingly, fetal bovine serum (FBS) in cell-culture medium functions as a stabilizer for surface protrusions, which reflects aspects of stabilizing function that are dependent on salt concentration^7^.

Neutralizing antibodies bind directly to the virus surface to inhibit attachment to host cells or otherwise interfere with entry mechanisms utilized by the virus. Thus, many studies on NNV vaccine development have focused on inducing higher titers of NNV-neutralizing antibodies because vaccine effectiveness is considered to correlate with those titers^2,3,16–24^. Recently, efficient methods have been developed to induce convalescence in fish after NNV infection^25–28^ and interestingly, despite the fact that convalescent fish were strongly protected from re-infection by NNV, almost no NNV-neutralizing antibodies were detected^29^. This suggests that there may be differences in the antigenicity of NNV *in vivo* compared to *in vitro*.

Different amino-acid regions of the P-domain have been identified as NNV neutralizing-antibody binding sites^12,22,30^. There have also been studies describing cellular receptors involved in attachment of NNV particles, and these include a sialic acid moiety on a glycan on the SSN-1 cell surface^31^, grouper heat shock cognate protein 70 (GHSC70) of GF-1 cells^32^, and an immunoglobulin-like cell adhesion molecule, nectin-4 of sevenband grouper^33,34^. In contrast, little is known about the receptor binding sites on NNV surface protrusions, so there has been no evidence that NNV-neutralizing antibody binding sites also function as receptor binding sites.

In this study, purified NNV particles were treated with different biochemical conditions to investigate functional alterations to antigenicity and infectivity. Using *in vitro* approaches our data allow us to discern that conformational stability and the positional relationship of NNV-neutralizing antibody binding sites and receptor binding sites are highly likely to be distinct.

## Results and discussion

**Figure 1:**
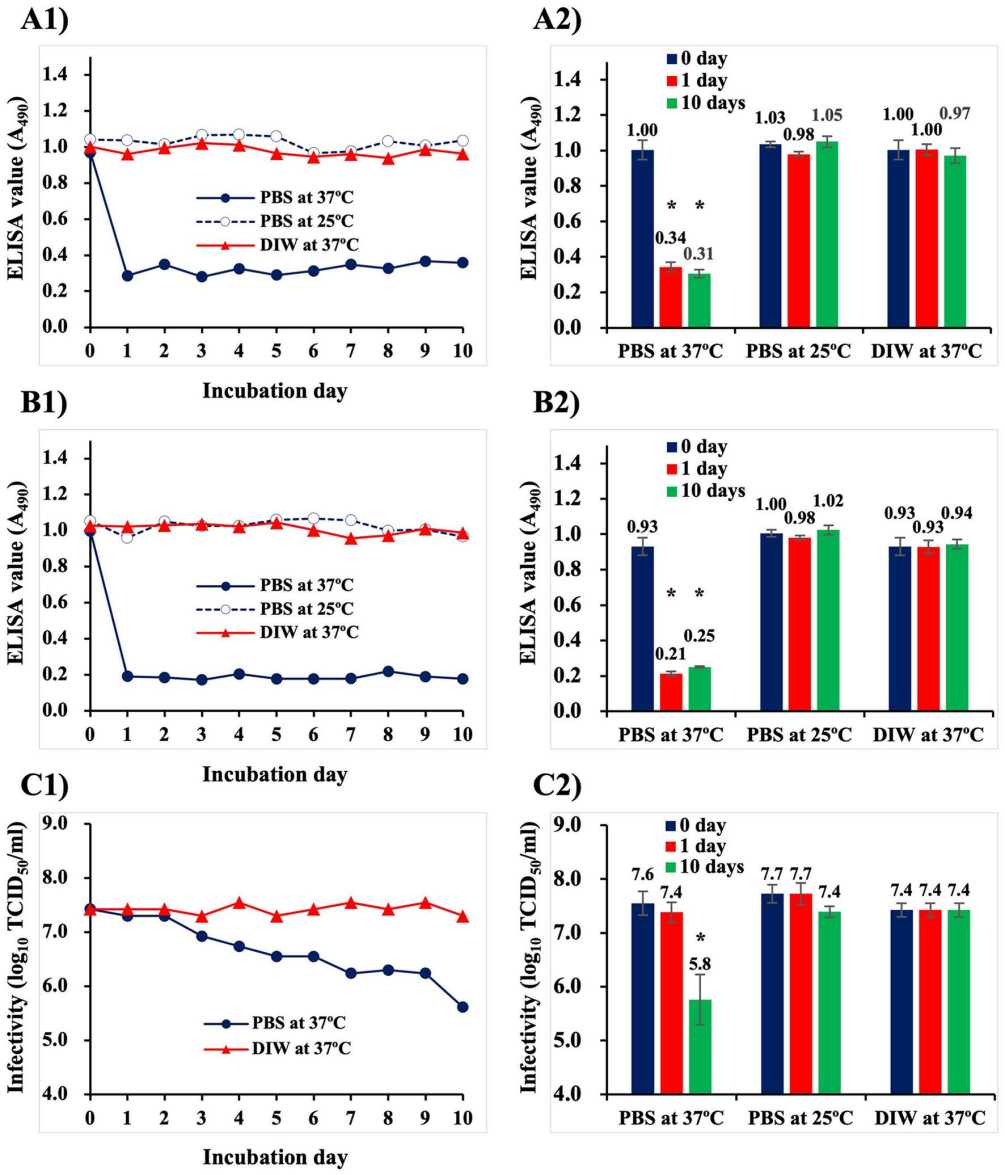
Alteration in antigenicity and infectivity of purified NNV particles after incubation at 37 °C in PBS. Purified NNV particles were incubated in PBS at 37 °C or at 25 °C, and in DIW at 37 °C for 10 days. (**A**) Alteration of NNV antigenicity detected with ELISA using anti-NNV PAb, (**B**) ELISA detection using anti-NNV MAb, (**C**) Alteration of NNV infectivity. (1) Results of a single set of experiments using the same sample on the same day. (2) The same experiments were conducted five times on different days, but limited to 0, 1 and 10 days incubation. Error bars indicate standard deviation (SD). *: Significant difference (ρ < 0.05) compared to the values on day 0.

It has been reported that both infectivity and antigenicity of purified NNV declines after incubation at 45 °C due to denaturation of NNV surface protrusions^7^. Interestingly, there was a difference between the time it took to lose antigenicity versus the length of time to lose infectivity (12 h and 24 h, respectively). In order to observe more detailed behaviors of surface protrusions to heat-denaturation, purified NNV particles immobilized on plate wells of enzyme-linked immunosorbent assay (ELISA) were treated with Dulbecco’s phosphate buffered saline (PBS) at 37 °C for 10 days. Treatments with PBS at 25 °C and deionized water (DIW) at 37 °C were also performed as temperature and buffer controls (Fig. 1A,B). Data shown in the left column of Fig. 1A1,B1 are the results from a single set of experiments at the same time. Regardless of detection with either anti-NNV rabbit serum (PAb) (Fig. 1A1) or anti-NNV mouse monoclonal antibody (MAb) (Fig. 1B1), ELISA values declined drastically by one day after incubation in PBS at 37 °C. Thereafter, no significant alteration was observed in those ELISA values. In contrast, no reduction was observed in ELISA values of NNV treated with either PBS at 25 °C or DIW at 37 °C for 10 days. Reproducibility of these experiments was assessed by repeating five times on different days (Fig. 1A2,B2), but were limited to treatments of zero, one and 10 days because the declination rate of ELISA values were drastically changed before and after the 1 day mark. It was confirmed that ELISA values of NNV declined one day after incubation at 37 °C in PBS from 1.00 to 0.34 (anti-NNV PAb) (Fig. 1A2, red bar) or from 0.93 to 0.21 (anti-NNV MAb) (Fig. 1B2, red bar). However, no further change was seen in the next nine days of incubation (Fig. 1A2,B2, green bars). The most likely explanation for the reduction in antigenicity is due to heat-denaturation, moreover, NNV surface protrusions are more heat-sensitive in PBS than DIW.

In order to observe alterations in NNV infectivity, purified NNV were treated with PBS or DIW at 37 °C for 10 days (Fig. 1C). The NNV infectivity in PBS at 37 °C declined gradually over 10 days, whereas no declination of infectivity was observed in DIW at 37 °C (Fig. 1C1). Although changes to NNV infectivity in PBS at 25 °C were not shown in this study, it has been reported that NNV are quite stable^7^. Also in testing reproducibility for NNV infectivity, no significant alteration was observed after one day of incubation in PBS at 37 °C, whereas it declined gradually from 10^7.6^ TCID_50_/ml to 10^5.8^ TCID_50_/ml over the next nine days (Fig. 1C2). These results suggest that NNV infectivity declined at a dramatically different rate than was seen for antigenicity under the same conditions.

**Figure 2:**
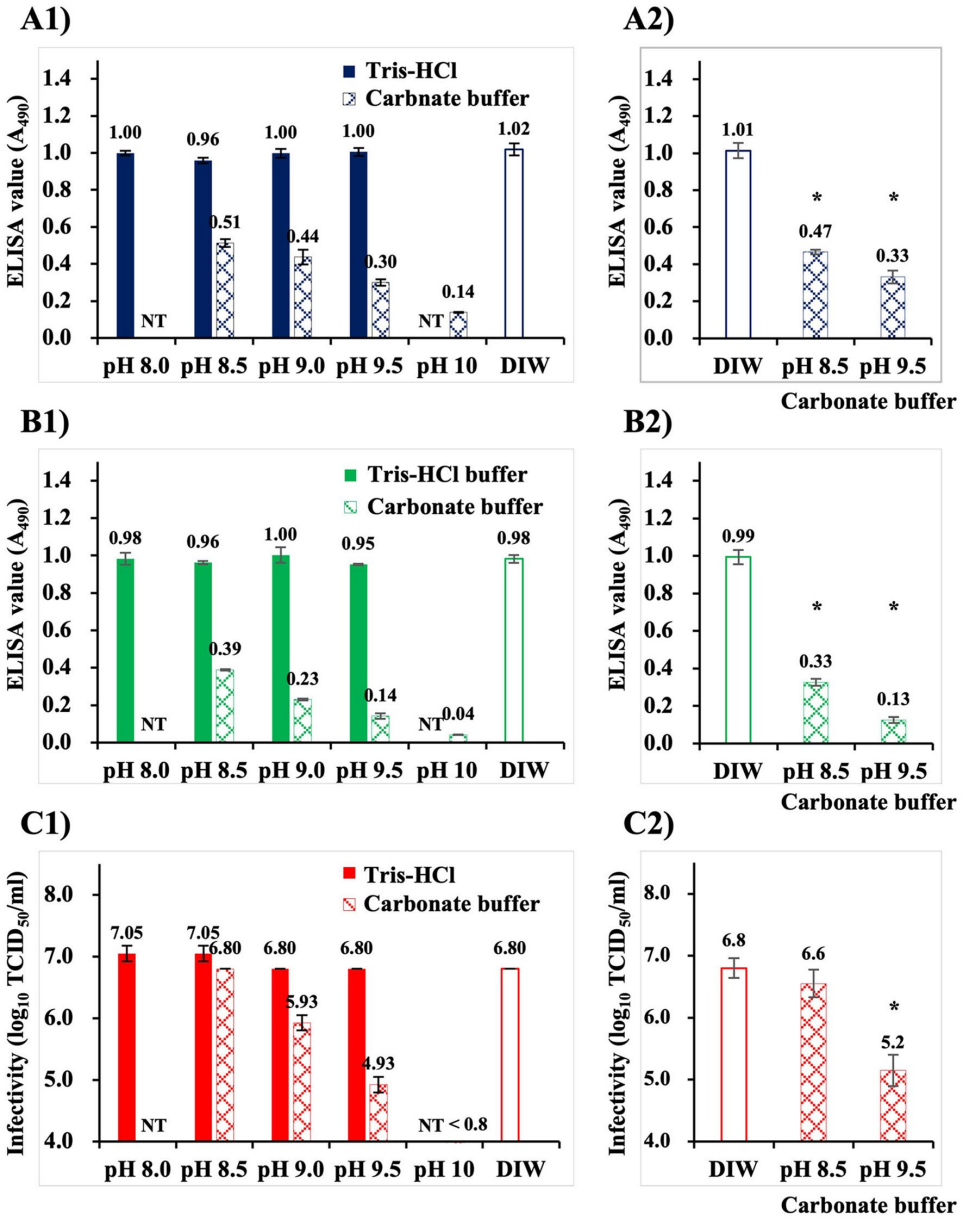
Alteration in antigenicity and infectivity of purified NNV particles after treatment with Tris-HCl or carbonate buffers. Purified NNV particles were treated with 15 mM Tris-HCl buffers (pH 8.0–9.5) or 100 mM carbonate buffers (pH 8.5–10.0) at 25 °C for 24 h. (**A**) Alteration of NNV antigenicity detected with anti-NNV PAb, (**B**) detection with anti-NNV MAb, (**C**) alteration of NNV infectivity. (1) Results of a single set of experiments using the same sample on the same day. (2) The same experiments were conducted five more times on different days, but limited to treatments with carbonate buffers at pH 8.5 and 9.5 and DIW. Error bars indicate SD. *: Significant difference (ρ < 0.05) compared to the values for the DIW treatment. NT: not tested due to out of an effective buffer range.

Recently, we demonstrated that the antigenicity and infectivity of NNV particles declined following treatment with carbonate buffer (pH 9.6) due to denaturation of NNV surface protrusions, but not with Tris-HCl buffer (pH 9.6)^15^. Thus, in order to observed more detailed behaviors on the denaturation of surface protrusions, purified NNV were treated with Tris-HCl buffers (pH 8.0–9.5) or carbonate buffers (pH 8.5–10.0), and were then assayed using anti-NNV PAb and MAb (Fig. 2A1,B1, a single set of experiments). When treated with Tris-HCl (pH 8.0–9.5), no alteration was observed in either ELISA values or infectivity of NNV (Fig. 2A1,B1,C1, closed bars). In contrast, ELISA values of NNV were halved or more after treatments with carbonate buffers (Fig. 2A1,B1, meshed bars). Also, NNV infectivity declined with increasing pH of carbonate buffers, except at pH 8.5 (Fig. 2C1, meshed bars). Five replicates were conducted but were limited to carbonate buffers at pH 8.5 and pH 9.5, and DIW (control) (Fig. 2A2,B2,C2). These pH conditions exhibited the maximum and minimum differences in antigenicity and infectivity between carbonate and Tris-HCl buffer treatments. ELISA values detected with PAb declined from 1.01 to 0.47 and 0.33 at pH 8.5 and pH 9.5, respectively (Fig. 2A2), while those detected with MAb declined from 0.99 to 0.33 and 0.13 at pH 8.5 and pH 9.5, respectively (Fig. 2B2). In contrast, no significant difference was observed in NNV infectivity between the treatments with carbonate pH 8.5 and DIW (10^6.6^ and 10^6.8^ TCID_50_/ml, respectively), whereas treatment at pH 9.5 was significantly lower (10^5.2^ TCID_50_/ml) than with DIW (Fig. 2C2). The observed declination in both of antigenicity and infectivity was likely due to the influence of carbonate concentrations, not due to pH, as has previously been described^15^. This conclusion is also supported by Tris-HCl results because, no reductions in antigenicity and infectivity of NNV were observed, regardless of pH (Fig. 2).

**Figure 3:**
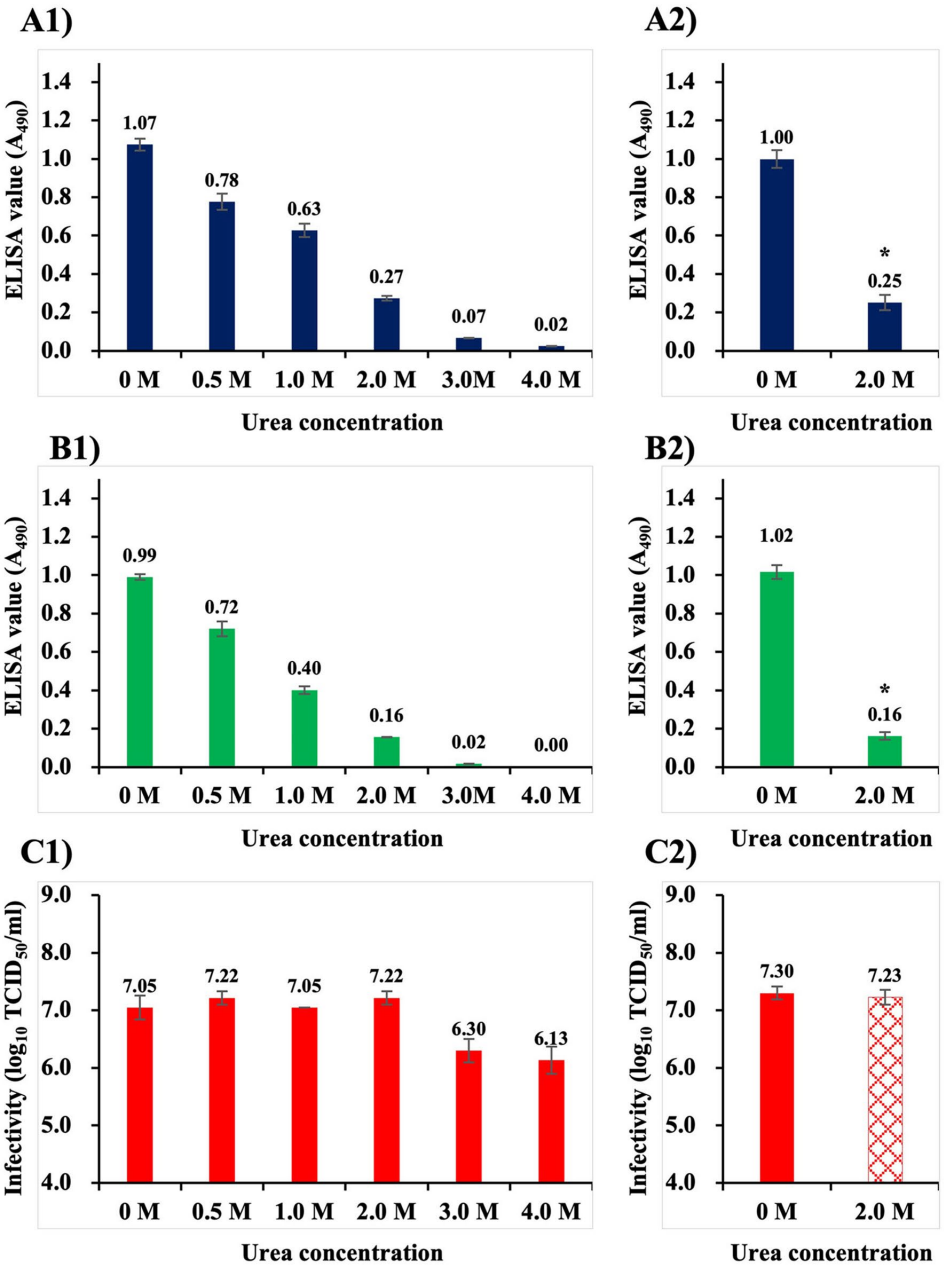
Alteration in antigenicity and infectivity of NNV particles following treatments at different urea concentrations. Purified NNV particles were treated with urea solutions at 25 °C for 24 h. (**A**) Alteration of NNV antigenicity detected with anti-NNV PAb, (**B**) detection with anti-NNV MAb, (**C**) alteration of NNV infectivity. (1) Results of a single set of experiments using the same sample on the same day. (2) The same experiments were conducted five times on different days, but limited to treatments at 0 and 2.0 M urea. Error bars indicate SD. *: Significant difference (ρ < 0.05) compared to the values for 0 M urea treatment.

As demonstrated above, antigenicity and infectivity of NNV appeared to decline at very different rates. Our future interest lies in identifying the sites on surface protrusions involved in NNV infection, meaning that a biochemical treatment that reduces only antigenicity without affecting infectivity is required. After studying various biochemical treatments, we arrived at urea, which is a protein denaturant for disrupting noncovalent bonds in proteins. Thus, in the next experiments, purified NNV particles were treated with urea solutions (0, 0.5, 1.0, 2.0, 3.0 and 4.0 M), and then detected with ELISA using anti-NNV PAb and MAb (Fig. 3A1,B1, a single set of experiments). ELISA values of NNV declined with increasing concentration of urea, regardless of which antibody was used (Fig. 3A1,B1). In addition, it was confirmed that NNV particles fixed on ELISA plate wells were not lost by treatment with urea at ≤ 4.0 M (see Supplementary Fig. S1 online). No declination was observed in infectivity of NNV treated with urea at ≤ 2.0 M (≥ 10^7.1^ TCID_50_/ml), whereas those treated with urea at ≥ 3.0 M were slightly lower (≤ 10^6.3^ TCID_50_/ml) (Fig. 3C1). Reproducibility tests were performed five times, and limited to zero and 2.0 M urea, the maximum concentration that was found not to affect NNV infectivity (Fig. 3A2,B2). It was confirmed that ELISA values of NNV particles declined from 1.00 to 0.25 (using PAb, Fig. 3A2) and to 0.16 (using MAb, Fig. 3B2), however, no alteration was observed in NNV infectivity after treatment with 2.0 M urea (Fig. 3C2). It was concluded that NNV infectivity was maintained following treatment with up to 2.0 M urea, but its antigenicity declined.

**Figure 4:**
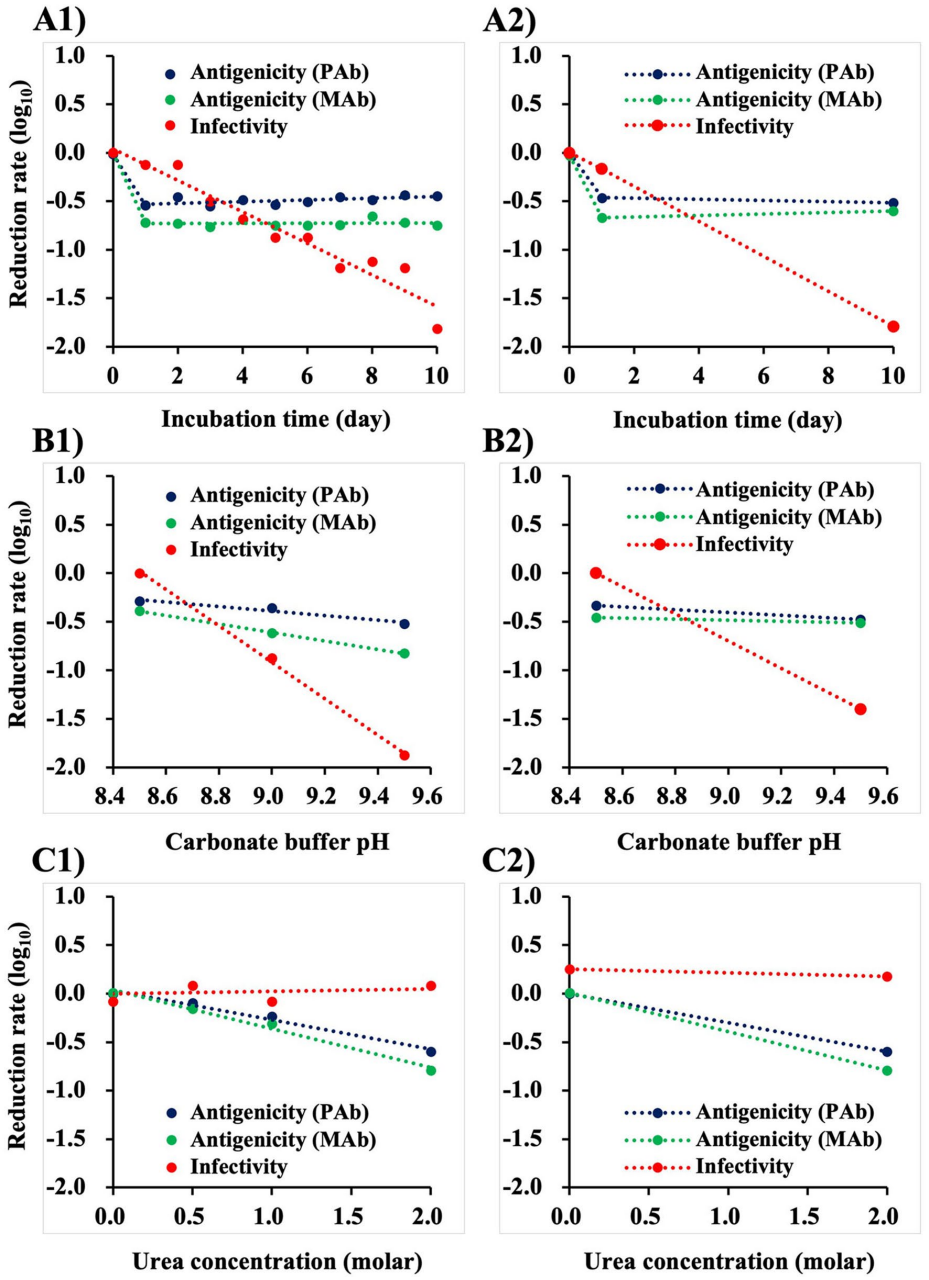
Comparison of declination patterns among NNV antigenicity and infectivity. The declination rate against control (treatment with DIW) were recalculated and are shown in semi-logarithmic graphs. (**A**) NNV incubated in PBS at 37 °C, (**B**) NNV treated with carbonate buffers at different pHs ranging from 8.5 to 9.5, (**C**) NNV treated with different concentration of urea (0 to 2.0 M). (1) Based on the results of a single set of experiments, (2) based on the reproducibility tests with five separate experiments.

The declining rate of NNV antigenicity and infectivity against controls were recalculated on semi-logarithmic plots (Fig. 4A,B,C). Figures in the left column were based on the results of a single set of experiments, while those in right column were based on the reproducibility tests with five repeated experiments. In addition, no significant difference was observed in the patterns of NNV antigenicity and infectivity between the left and right columns. NNV infectivity declined much more drastically than NNV antigenicity under conditions of both PBS at 37 °C (Fig. 4A) and carbonate buffer (Fig. 4B). In contrast, NNV antigenicity declined in the treatments with ≤ 2.0 M urea, but infectivity was maintained (Fig. 4C). These results demonstrate that antigenicity and infectivity of NNV behaved independently and were dependent on the nature of biochemical treatments.

Both anti-NNV PAb and the MAb used in this study have high titers of NNV-neutralization, and recognize heat-sensitive conformational epitopes on surface protrusions^7,10^. Thus, the observed declination in NNV antigenicity was likely due to denaturation of conformational epitopes on protrusions. Chen *et al.*^6^ suggested several amino-acid stretches of CP including aa 223–227, aa 233–237, aa 253–259 and aa 285 − 291, as potential epitopes for immunoreactivity of NNV serotype C, as these sequences are located on the surface of protrusions and diverge between different NNV serotypes. Panzarin *et al.*^12^ reported that an antiserum neutralizing NNV serotype C recognized aa 217–256 region based on analysis using chimeric CPs of serotypes A and C, while Lin *et al.*^22^ reported that a five tandem-repeated from aa’s 249–258 generated NNV-neutralizing antibodies *in vivo*. Costa *et al.*^30^ reported that the aa 181–212 region was recognized by neutralizing MAb of NNV serotype C based on peptide-scan analysis, although this was not within the stretch for the protrusion domain region described by Chen *et al.*^6^. These previous studies suggested that there could be multiple epitopes for binding with NNV-neutralizing antibodies. Of them, one or more conformational epitopes on surface protrusions might be recognized by the PAb and MAb used in this study because both also recognize conformational epitopes on protrusions involved with virus neutralization^7,10^. Although epitopes recognized by the PAb and MAb we used have not so far been identified, a competition ELISA revealed that the epitope recognized by the MAb is one of those recognized by the PAb^10^. It is quite possible that the PAb contains antibodies recognizing epitopes not involved with viral neutralization. However, no significant difference was observed in the altered patterns of NNV antigenicity between either PAb or MAb (Fig. 4). This suggests that NNV antigens detected with the PAb could be mainly due to antibodies recognizing epitopes involved with viral neutralization.

Attachment of viral particles to host cells via cellular receptors is the first critical step in infection^35^. As described above, antigenicity and infectivity of NNV particles behaved quite differently in the face of different biochemical treatments. Depending on the condition, it was possible to denature the conformational epitopes for binding with NNV-neutralizing antibodies, while maintaining NNV infectivity (incubation at 37 °C in PBS for 1 day, treatment with carbonate buffer pH 8.5, treatment with 2.0 M urea, Figs. 1, 2, 3 and 4). The maintenance of infectivity means that receptor binding sites on NNV surface protrusions are also maintained. Therefore, it appears likely that the neutralizing antibody binding sites and receptor binding sites are located independently on surface protrusions. Furthermore, in the face of urea treatments, the receptor binding sites could be more structurally stable than the antibody binding sites (Fig. 3). Interestingly, Ito *et al*^9^ reported that the CP regions encompassing amino acid stretch aa 223–244 (encoded in RNA2 nt 649–758) was required for NNV serotype C to determine its host fish species. This region overlaps with that for NNV-neutralizing antibody binding sites described by Panzarin *et al.*^12^. However, SSN-1 cells are susceptible to all NNV serotypes^8^. There has been no evidence that NNV CP, rather than the viral genome, controls host specificity. Thus, the same authors also suggested that there might be another site on surface protrusions that binds receptors on SSN-1 cells^9^. This is consistent with our conclusion that NNV-neutralizing antibody binding sites recognized by the PAb and MAb are probably independent from receptor binding sites.

It was noted that of the tested biochemical treatments, exposure to 2.0 M urea was the best at significantly reducing NNV antigenicity while maintaining receptor binding sites on NNV surface protrusions. We believe that NNV treated with 2.0 M urea could be suitable for identifying the receptor binding sites. Recently, Liu *et al.*^31^ revealed that the sialic acid moiety of glycans on the surface of SSN-1 cells functions as a receptor for NNV. Sialic acid linked to glycoproteins and gangliosides is used by many viruses as receptors^36,37^. In order for positional and structural analyses of receptor binding sites on NNV protrusions, it would be necessary to identify sialic acid linked receptors on host cells including SSN-1.

## Methods

### Virus culture and purification

NNV SgNag05 (RGNNV genotype, serotype C) was cultured with SSN-1 cells at 25 °C. SSN-1 cells were maintained in Leibovitz’s L-15 medium (Gibco) containing 10% (v/v) FBS (Gibco), 150 IU/ml of penicillin G, and 100 μg/ml of streptomycin. NNV particles were purified following methods described previously^7,38,39^. Briefly, after centrifugation (12,000 × *g*, 20 min, 4 °C), the resulting NNV suspension was dialyzed in PBS, 15 mM Tris–HCl (pH 8.0) and DIW for one, three and one days, respectively, using Biotech cellulose ester (CE) membrane tube with a molecular weight cut off (MWCO) of 10^6^ (Spectrum Laboratories). The dialyzed NNV suspension was subjected to anion-exchange chromatography using a Hi-trap Q column (GE Healthcare). NNV particles eluted with 700 mM NaCl were desalinated by dialysis in DIW for 1 day, using Biotech regenerated cellulose (RC) tube at 1.4 × 10^4^ MWCO (Spectrum Laboratories). The resulting NNV suspension was subjected to centrifugal ultrafiltration at 3 × 10^5^ MWCO (Vivaspin, Sartorius) to obtain monomeric NNV particles as described previously^39^.

### NNV infectivity experiments

In order to observe changes to infectivity, purified NNV particles were suspended in: (1) 15 mM Tris–HCl (pH 8.0), PBS or DIW, and incubated at 37 °C or 25 °C for 10 days; (2) NNV particles were suspended in 0, 0.5, 1.0, 2.0, 3.0 or 4.0 M urea solution, and incubated at 25 °C for 24 h; and (3) NNV particles were suspended in 100 mM carbonate/bicarbonate buffers (pH 8.5, 9.0, 9.5 and 10.0), 15 mM Tris–HCl buffers (pH 8.0, 8.5, 9.0 and 9.5) or DIW, and incubated at 25 °C for 24 h. NNV infectivity was titrated using 96-well microplates seeded with SSN-1 cells. Appearance of cytopathic effect (CPE) was evaluated to determine 50% tissue culture infectious dose (TCID_50_) after 10 days of culture at 25 °C.

### NNV antigenicity experiments

It has been reported that the antigenicity of NNV particles depends on salt concentrations due to changes in viral aggregation state^7,39,40^. Therefore, NNV particles were resuspended in DIW and immobilized onto wells of ELISA plates (Greiner Bio-One) by drying at 37 °C overnight, followed by treatment with different buffers under the same conditions described above (Section “NNV infectivity experiments”). The treated NNV particles were detected with PAb, MAb (14D11), horseradish peroxidase (HRP) conjugated antisera against rabbit or mouse Ig (Dako) and OPD substrate solution (1 mg/ml *ο*-phenylenediamine, 0.03% H_2_O_2_, 100 mM Na_2_HPO_4_, 50 mM citric acid). ELISAs were performed according to previously published methods^7,15,39,41^.

### Statistical analysis

ELISA values and infectivity titers were analyzed using t-test. Statistical significance level was set at *p* < 0.05.

## Supplementary Information

Supplementary Figure S1.
