# Supplementary material for: Sites responsible for infectivity and antigenicity on nervous necrosis virus (NNV) appear to be distinct
Source: Sci Rep. 2021 Feb 11;11:3608. doi: 10.1038/s41598-021-83078-3 (PMC7878751; doi:10.1038/s41598-021-83078-3)
Supplement: Supplementary file 1 — Supplementary Figure S1. [file 41598_2021_83078_MOESM1_ESM.docx]

Sites responsible for infectivity and antigenicity on nervous necrosis virus (NNV) appear to be distinct

Hyun Jung Gye and Toyohiko Nishizawa

Department of Aqualife Medicine, Chonnam National University, Yeosu, Republic of Korea

**Supplementary figures with figure legends**


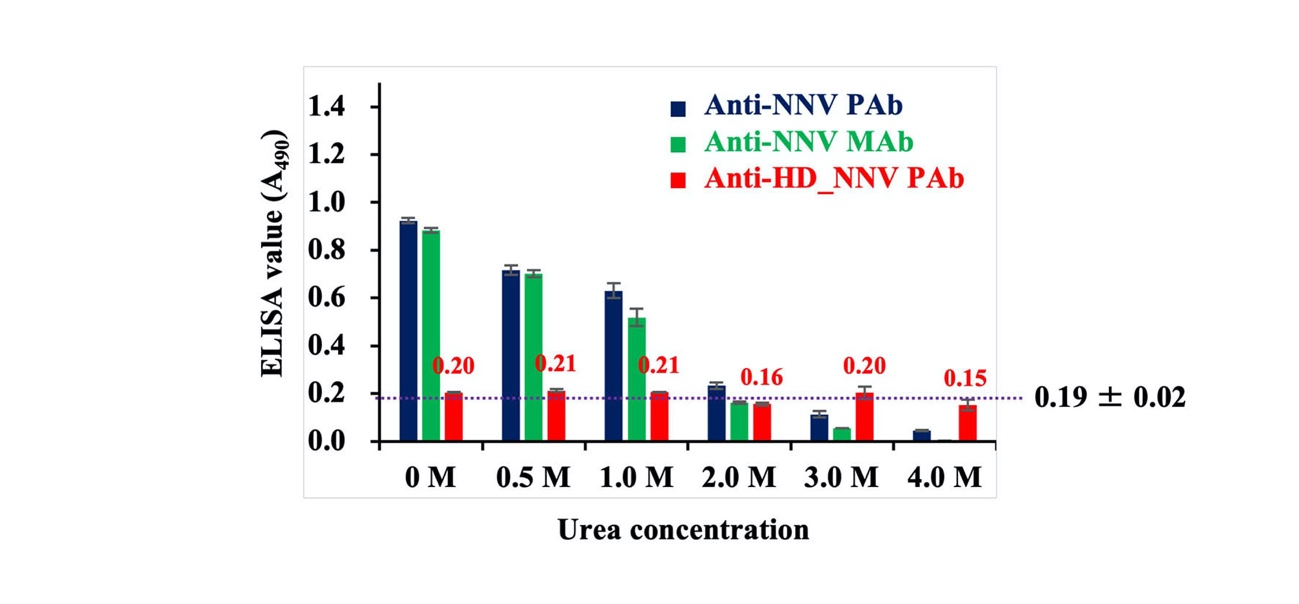


**Figure S1. Antigenicity of NNV particles treated with different concentrations of urea.** Following the same experiment performed in the same manner as seen in Figure 2, NNV antigens were detected not only with anti-NNV PAb and MAb, but also with anti-HD_NNV PAb. The anti-HD_NNV PAb contains antibodies against NNV particles treated at 100ºC for 5 min ^[10]^. Due to this treatment, conformational structures of surface protrusions on NNV particles were completely denatured, but viral particle structures were maintained ^[7]^. Thus, the anti-HD_NNV PAb recognizes NNV particles, but not NNV surface protrusions. ELISA values of NNV declined with increasing concentration of urea, regardless of detection with anti-NNV PAb or MAb (blue and green bars, respectively). Against this, no declination was observed in the ELISA values of NNV detected with anti-HD_NNV PAb even though the concentrations of urea increased (red bars). These results demonstrate that NNV particles fixed on ELISA plate wells were not lost by treatment with urea at up to 4.0 M, suggesting that the declination in ELISA values of NNV detected with anti-NNV PAb and MAb (blue and green bars) were due to denaturation of NNV surface protrusions.
